# Retraction: Improved cognition impairment by activating cannabinoid receptor type 2: Modulating CREB/BDNF expression and impeding TLR-4/NFκBp65/M1 microglia signaling pathway in D-galactose-injected ovariectomized rats

**DOI:** 10.1371/journal.pone.0303444

**Published:** 2024-05-03

**Authors:** The PLOS ONE Editors

After this article [1] was published, concerns were raised about results presented in Figs 4–7. Specifically:

- Fig 4C in [1] appears similar to Fig 5C in [2] and these panels are reported to represent different experimental conditions.
- In Fig 5 in [1] similarities were noted between the following panels reported to represent different experimental conditions:
  ○ Panel B appears similar to Fig 5B in [2].
  ○ The upper region of Fig 5B appears to have similar features to the lower region of Fig 5F when flipped.
  ○ Panel C appears similar to Fig 6C in [2].
  ○ Panel D appears similar to Fig 6E in [2] with the exception of an area in the center of the panels which appears different.
  ○ Panel G appears similar to Fig 6I in [2].
  ○ Panel H appears similar to Fig 5E in [2].
- In Fig 6 in [1] similarities were noted between the following panels reported to represent different experimental conditions:
  ○ Panel D appears similar to Fig 9F and 9H in [2] when rotated 90 degrees.
  ○ The upper region of panels d and p appear similar when rotated 180 degrees.
  ○ Panel j and panel f appear to show different magnifications of the same image.
  ○ Panel K appears similar to Fig 9A in [2].
  ○ Panel L appears similar to Fig 9B and 9J in [2] when rotated 90 degrees.
- In Fig 7:
  ○ Panels b and d appear similar when flipped.
  ○ There appear to be areas of overlap between panels h, j, l, n, and p.

In response to queries about the experiments in Figs 6 and 7, the corresponding author stated that Fig 6J and 6P and Fig 7B, 7H, 7N and 7P are incorrect due to an error. They provided underlying images for all panels in Figs 4–8 and individual-level underlying data for the charts in Figs 2, 3 and 5–8, but the *PLOS ONE* Editors did not consider this to resolve concerns about the validity of the image data.

In light of the extent of the concerns listed above that question the reliability and validity of the reported results and conclusions, the *PLOS ONE* Editors retract this article.

SSAER and HMF agreed with the retraction and apologize for the issues with the published article.

Owing to the concerns about similarities with previously published content [2], published in 2021 by The Pharmaceutical Society of Japan [2], which is not offered under a CC BY license, Figs 4C, 5B–5D, 5G–5H, 6D and 6K–6L are excluded from this article’s [1] license. At the time of retraction, the article [1] was republished to note these exclusions in the legends of Figs 4–6 and the article’s copyright statement.
